# Are Argonaute-Associated Tiny RNAs Junk, Inferior miRNAs, or a New Type of Functional RNAs?

**DOI:** 10.3389/fmolb.2021.795356

**Published:** 2021-12-03

**Authors:** Kotaro Nakanishi

**Affiliations:** ^1^ Department of Chemistry and Biochemistry, The Ohio State University, Columbus, OH, United States; ^2^ Center for RNA Biology, Columbus, OH, United States

**Keywords:** argonaute, dicer, tiny RNA, miRNA, tRNA, siRNA, miRNA trimming, miRNA degradation

## Abstract

The biosynthesis pathways of microRNAs (miRNAs) have been well characterized with the identification of the required components. miRNAs are synthesized from the transcripts of miRNA genes and other RNAs, such as introns, transfer RNAs, ribosomal RNAs, small nucleolar RNAs, and even viral miRNAs. These small RNAs are loaded into Argonaute (AGO) proteins and recruit the effector complexes to target mRNAs, repressing their gene expression post-transcriptionally. While mature miRNAs were defined as 19–23 nucleotides (nt), tiny RNAs (tyRNAs) shorter than 19 nt have been found to bind AGOs as equivalent or lesser miRNAs compared to their full-length mature miRNAs. In contrast, my recent study revealed that when human AGO3 loads 14 nt cleavage-inducing tyRNAs (cityRNAs), comprised of the first 14 nt of their corresponding mature miRNA, it can become a comparable slicer to AGO2. This observation raises the possibility that tyRNAs play distinct roles from their mature form. This minireview focuses on human AGO-associated tyRNAs shorter than 19 nt and discusses their possible biosynthesis pathways and physiological benefits, including how tyRNAs could avoid target-directed miRNA degradation accompanied by AGO polyubiquitination.

## Introduction

**FIGURE 1:**
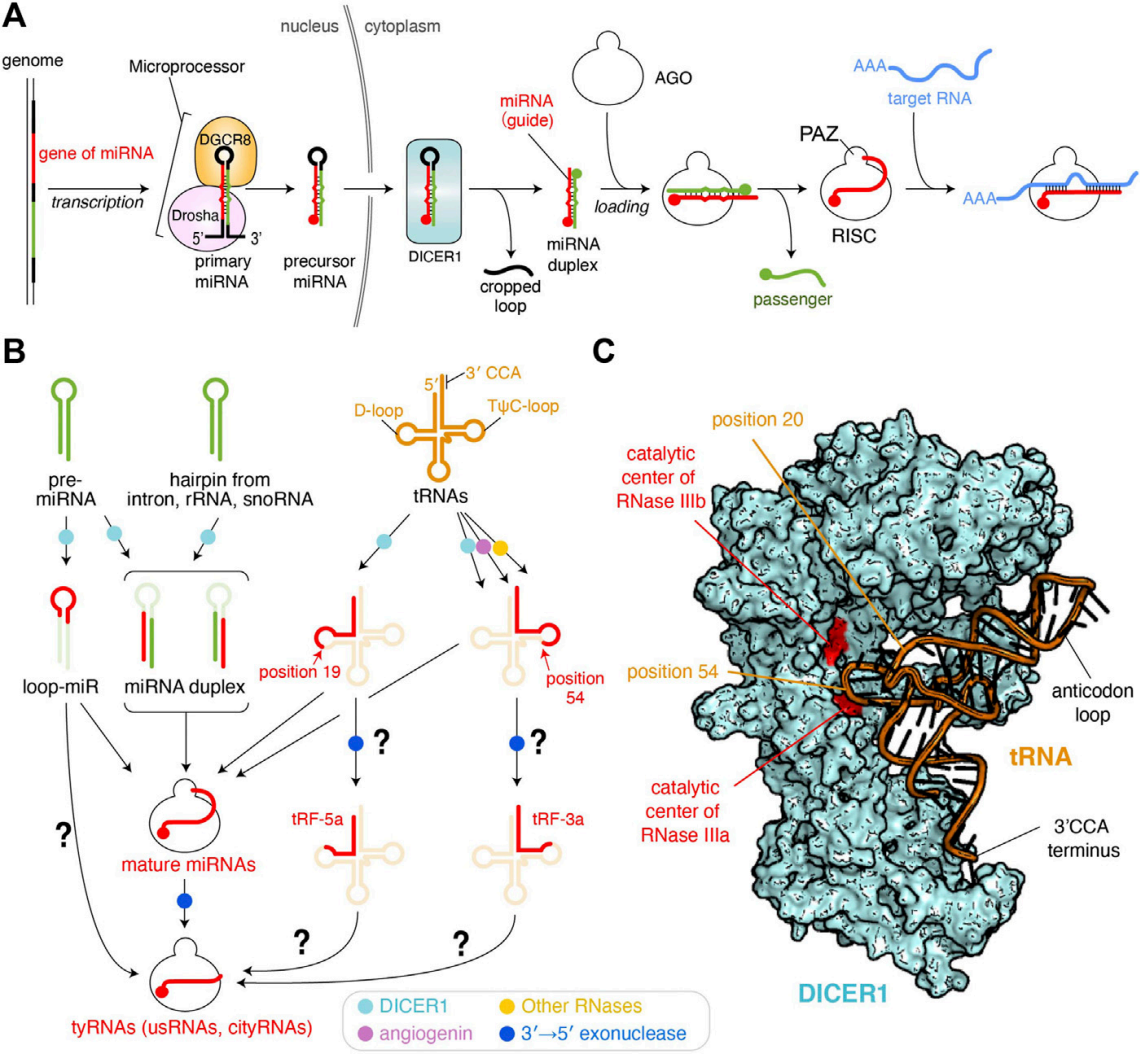
Biogenesis pathways of miRNAs and tyRNAs. **(A)** Canonical miRNA biogenesis. 5′ monophosphate groups are depicted as spheres. The schematic is modified based on the figure in the previous review (Nakanishi, 2016) **(B)** Biogenesis of tyRNAs derived from miRNAs and other RNAs. Enzymes involved in the pathway are shown as different colored dots. The pathways to be studied further are shown with a question mark. **(C)** Model of tRNA-docked onto human DICER1. The DICER1 structure of the original coordinates (PDB: 5ZAL) reflects an inactive form because the C-terminal double-stranded RNA-binding domain (dsRBD) interferes with binding of the double-stranded RNA to the RNase IIIa and IIIb. The dsRBD is moved away to make a slicer-active model. A tRNA (PDB ID: 1EHZ) is manually docked.

More than 2,000 microRNAs (miRNAs) have been reported in humans as of 2019 (Kozomara et al., 2019). miRNAs are varied in sequence, but their lengths fall within a range of 19–23 nucleotides (nt) because precursor miRNAs (pre-miRNAs) are processed by Dicer, a molecular ruler which generates size-specific miRNA duplexes (Zhang et al., 2004). After these duplexes are loaded into Argonaute (AGO) proteins, one strand is ejected while the remaining guide strand and AGO form the RNA-induced silencing complex (RISC) (Figure 1A) (Nakanishi, 2016). Therefore, a length of about 22 nt is the hallmark of mature miRNAs (Ambros et al., 2003). This size definition was exploited to eliminate ∼18 nt RNAs during sample preparation or analysis in most early next-generation RNA sequencing (RNA-seq). However, RNA-seq without this limitation found a substantial number of 10–18 nt tiny RNAs (tyRNAs) bound to AGOs (Gangras et al., 2018; Kuscu et al., 2018; Baldrich et al., 2019). This review defines AGO-associated 19 nt or longer small RNAs as mature miRNAs, with those shorter than 19 nt as tyRNAs. Although these tyRNAs have been reported to retain a similar physiological activity to their mature miRNAs, some of 14–15 nt tyRNAs drastically increase the slicing activity of human AGO3 and named them cleavage-inducing tyRNAs (cityRNAs) (Park et al., 2020). The discovery has raised the possibility that tyRNAs confer yet-unidentified, but distinct roles on AGOs.

## Precursors of Tiny RNAS

There have been only a few papers reporting tyRNAs and characterizing their physiological activity. The previous studies show that tyRNAs are derived from various types of RNAs.

### tRNA-Derived tyRNAs

Transfer RNAs (tRNAs) are mediator molecules decoding genomic information into amino acids. As one of the most indispensable noncoding RNAs throughout evolution, tRNAs have been involved in and responsible for many different cellular events (Su et al., 2020). Previous studies revealed that angiogenin is induced to cleave tRNAs within anticodon loops upon starvation, ultraviolet irradiation, oxidative stress, heat/cold stress, and hypoxia (Thompson and Parker, 2009; Yamasaki et al., 2009). Human DICER1 was also reported as a tRNA slicer (Figure 1B) (Cole et al., 2009), but meta-analysis suggests that the generation of tRNA-derived fragments (tRFs) is DICER1-independent because more tRFs were generated in dicer −/− mouse embryonic stem cells (Kumar et al., 2014). Although DICER1 is dispensable for the generation of most of 15–30 nt tRFs, tRNA^Trp^(CCA), tRNA^His^(GTG), and tRNA^Tyr^(GTA) show 2–10-fold down-regulation of their 15–19 nt fragments in the dicer1 knockout cells (Li et al., 2012), suggesting that DICER1 is required for the synthesis of 15–19 nt tRFs. Indeed, recombinant human DICER1 cleaves a body-labeled tRNA^Gln^ to 20–30 nt *in vitro* (Cole et al., 2009). Also, DICER1 processes tRNA^Gly^ in its TψC-loop to generate a 22 nt small RNA called CU1276, which works as a miRNA (Maute et al., 2013).

Despite the strong evidence of tRNA cleavage by DICER1, how DICER1 recognizes tRNAs to generate tRFs remains unclear. In canonical miRNA biogenesis, human DICER1 recognizes the 3′ end 2 nt overhang of pre-miRNA and with its RNase III domains cleaves the position about 22 nt from the 3′ end (Park et al., 2011). Also, DICER1 generates size-defined products from two pre-miRNAs, one of which has a 34 nt stem and a 14 nt loop while the other has a 22 nt stem and a 4 nt loop (Tsutsumi et al., 2011), indicating that DICER1 measures 22 nt from the 3′ end and cleaves the pre-miRNA, regardless of stem and loop length. Likewise, if DICER1 recognizes the 3′ end 4 nt overhang of L-shaped tRNAs with its PAZ domain, where does DICER1 cleave in the tRNAs? When a tRNA is docked on the structure of human DICER1 (Liu et al., 2018), the phosphate backbone at position 54 on the TψC-loop is in the vicinity of the catalytic residues of the RNase IIIa domain (Figure 1C). This is consistent with the cleavage site of tRNA^Gly^ by DICER1 (Maute et al., 2013). An RNA sequencing analysis showed that 19 nt tRFs are the most abundant and are typically generated by cleavage at the phosphate group of position 20. In the docking model, the phosphate group at position 20 in the D-loop appears to be accessible to the catalytic centers of neither RNase IIIa nor IIIb (Figure 1C). Still, the scissile phosphate group appears to face the catalytic residues if the L-shaped tRNA is unwound by DICER1.

tRFs are classified into 10 categories (Xie et al., 2020; Zong et al., 2021). Among them, 14–30 nt tRF-5s were found in AGO1- and AGO2-associated small RNAs in HeLa cells, and their production required DICER1 (Cole et al., 2009). tRF-5s are further classified into three subgroups, one of which is the 14–16 nt tRF-5a generated from a region from the 5′ end of the acceptor stem to the D-loop or its stem part (Figure 1B) (Kumar et al., 2014). Meanwhile, tRF-3s, which are derived from a region from TψC-loop to the 3′ CCA terminus, are known to be incorporated into AGO1 and AGO2, and their synthesis involves angiogenin, DICER1, and other RNases (Li et al., 2012). tRF-3a, a subtype of tRF-3s, is ∼18 nt. It may be plausible that after being cleaved by DICER1, tRF-5 and tRF-3 are loaded into AGO and then processed into 14–18 nt tRFs by a 3′→5′ exonuclease (Figure 1B).

### Unusually Small RNAs

The genomic DNA and RNA of viruses, such as Epstein-Barr virus, bovine leukemia virus, and Kaposi’s sarcoma-associated herpesvirus (KSHV), encode their miRNA genes (Gottwein et al., 2007; Kincaid et al., 2012; Pfeffer et al., 2004). In the infected cells, the viral transcript, mimicking primary miRNAs, are processed by Microprocessor in the nucleus and/or DICER1 in the cytoplasm (the process corresponds to that of the cellular pri-miRNAs in Figure 1A) (Kincaid and Sullivan, 2012). After the resultant viral miRNA duplexes are loaded into AGO, the hijacked RISCs manipulate mRNAs relevant to the antiviral response and apoptosis to increase the infected cell’s longevity (Abend et al., 2010; Suffert et al., 2011). For example, human cytomegalovirus miR-UL112 targets the gene of the major histocompatibility complex class I-related chain B, which is a stress-induced ligand of natural killer cells and actives the receptor to kill virus-infected cells (Stern-Ginossar et al., 2007).

KSHV generates 12 viral miRNAs (Grundhoff et al., 2006). Northern blots for 22 nt K12-1 KSHV miRNA also detected a 16 nt RNA, whose sequence is identical to the first 16 nt of the 22 nt form (Li et al., 2009). This unusually small RNA (usRNA) silences the gene expression, albeit the activity is different from its mature form. The authors analyzed the previously reported RNA-seq data of AGO-associated small RNAs in HEK293 cells (Ender et al., 2008) and discovered cellular usRNAs whose sequence is identical to the first 18 nt of their miRNAs (Li et al., 2009). Since the MID and PIWI domains of human AGOs thoroughly recognize the 5′ end of the bound guide RNA (Elkayam et al., 2012; Nakanishi et al., 2012; Schirle and MacRae, 2012; Nakanishi et al., 2013; Park et al., 2017; Park et al., 2019), the 5′ end seems to have no chance of being processed by 5′→3′ exonucleases. On the other hand, the 3′ end of AGO-bound guide RNAs, which is recognized by the PAZ domain in the RISC, is known to be trimmed by different 3′→5′ exonucleases (Juvvuna et al., 2012; Yoda et al., 2013). In the paper, high-throughput sequencing showed that 9–14 nt RNAs were the most abundant usRNAs, but the population was not analyzed further due to the technical difficulty in reliably mapping such short reads to the genomic coordinates (Li et al., 2009). These results suggest that usRNAs are derived from AGO-bound mature miRNAs by 3′ end trimming, but RNases and components involved in the 3′ end trimming remain to be studied.

### Loop-miRs

In the canonical miRNA-biogenesis pathway, Dicer processes the hairpin-structured pre-miRNAs into miRNA duplexes while generating the cropped loops as a byproduct (Nakanishi, 2016) (Figure 1A). However, when the synthetic RNAs corresponding to the excised loop region were transfected into HEK293 cells, they repressed the luciferase activity (Winter et al., 2013), suggesting that the loop region works as guide RNA to silence the gene expression. Thus, the single-stranded loop region of pre-miRNA hairpins is named loop-miR (Figure 1B). According to the size definition of miRNAs, the loop parts of miR-33a (19 nt), miR-34a (20 nt), miR-192 (22 nt), and miR-219 (22 nt), but not any loops shorter than 19 nt, were tested for biological significance in the study. The loop excised by Dicer shows a size distribution mainly from 8 to 29 nt, with 13–15 nt being the most abundant (Winter et al., 2013). Although efficient loading of miRNA duplexes into AGOs requires chaperon machinery (Iwasaki et al., 2015; Naruse et al., 2018), 15–29 nt 5′ phosphorylated single-stranded RNAs can be autonomously incorporated into AGOs in HeLa cell cytoplasmic extract (Martinez et al., 2002). It looks like 13–15 nt loop-miRs are directly loaded into AGOs, but another study showed that when pre-miR-34 mutant has a 15 or 22 nt loop, the hairpin was processed properly regardless of the loop size but did not produce the 15 nt species loaded to fly Ago1 (Okamura et al., 2013). This study also reported that fly Ago1-loaded loop-miRs were trimmed at their 3′ end and that mouse AGO1 and AGO2 loaded shortened loop-miRs. Those results suggest that sufficient length loop-miRs are loaded into AGOs and processed into tyRNAs by 3′ end trimming.

## Benefits of Synthesizing Tyrnas to the Cells

Previous studies using RNA-seq and *in vivo* assays demonstrated that many tyRNAs exist in cells and bind to AGOs, and that these RNAs can cause gene silencing similar to their mature miRNAs. The advantages of synthesizing tyRNAs, however, remain unclear.

### cityRNA-Bound AGO3 Could Cleave Many mRNAs

In humans, AGO2 has been thought to be the only slicer when the targets have a fully complementary sequence to their guide RNA (Liu et al., 2004; Meister et al., 2004). siRNAs can be designed to perfectly pair any mRNAs and cleave them by exploiting AGO2’s slicing activity (Elbashir et al., 2001). Only a few endogenous RNAs, however, have been reported as the substrate of miRNA-directed mRNA cleavage (Lagos-Quintana et al., 2001). HOXB8 mRNA includes a sequence fully complementary to 22 nt miR-196a (Yekta et al., 2004). Since guide RNAs do not use the base at their guide nucleotide position 1 for pairing with target RNAs, a sequence perfectly complementary to a 22 nt guide appears every 1.1 trillion nucleotides (= 4^21^ = 4.4 × 10^12^). This number is quite large compared to the diploid human genome size of 6.4 billion nucleotides (6.4 × 10^9^). Therefore, few transcripts encompass the perfectly complementary sequence, which could explain why only a few mRNAs are cleaved by AGO2 with mature miRNAs. My recent study discovered that loading 14 nt specific tyRNA catalytically activates AGO3, while reducing the slicing activity of AGO2 compared to that of their mature miRNAs (Park et al., 2020). The sequence fully complementary to a 14 nt cityRNA appears every 67 million nucleotides (= 4^13^ = 67 × 10^6^). Assuming that about 93% of the human genome is transcribed (Consortium et al., 2007), AGO3 loaded with a 14 nt cityRNA could cleave approximately 100 sites. This number decreases because among 14–15 nt tyRNAs, only ones with specific sequences can serve as cityRNAs (i.e., catalytically activate AGO3) (Park et al., 2020). Nevertheless, the synthesis of cityRNAs appears to be a powerful tool for the cell to change gene expression drastically by cleaving many transcripts. Therefore, cells would need to regulate the biogenesis of tyRNAs strictly.

## Possible Pathways of Tiny RNA Biogenesis

### Direct Loading of tyRNAs Into AGOs

Canonical and most non-canonical miRNA biogenesis pathways, including intron-, ribosomal RNA-, and small nuclear RNA (snoRNA)-derived miRNA syntheses, retain the hairpin or double-stranded form of their precursors until the passenger strand is ejected during RISC assembly (Figure 1B) (Okamura et al., 2007; Ruby et al., 2007; Ender et al., 2008). In contrast, it remains unclear whether tRFs are loaded into AGOs as single- or double-stranded RNAs. Piwi-interacting RNAs (piRNAs) are known to originate from single-stranded RNA precursors, and the 3′ end of their mature form is protected by a 2′ O-methyl modification from RNA degradation (Czech et al., 2018; Ozata et al., 2019). Although little is known about how the single-stranded precursors of piRNAs retain the integrity during processing, they must have a yet-identified system to avoid RNA degradation.

If ∼18 nt single-stranded RNAs are generated in the cell, can they be directly loaded into AGOs as tyRNAs? Such short RNAs would be quite susceptive to cellular RNases because they are too short to fold into a hairpin structure or form a stable duplex. Supporting this idea, my recent study showed that transfection of an unmodified, 14 nt guide RNA into HEK293T cells failed to activate AGO3 (Park et al., 2020), probably due to single-stranded RNA degradation by the cellular RNases (Lima et al., 2012). However, when the same 14 nt single-stand was heavily modified, it loaded onto AGO3 and converted it to an active slicer. These results suggest that even if tyRNAs are endogenously synthesized in the cell, most are easily degraded before interacting with AGOs. How single-stranded precursors of miRNAs and piRNAs protect themselves from degradation, and whether they employ similar or distinct mechanisms, remains to be studied.

### tyRNAs May Escape From Trimming- and Tailing-independent TDMD

**FIGURE 2:**
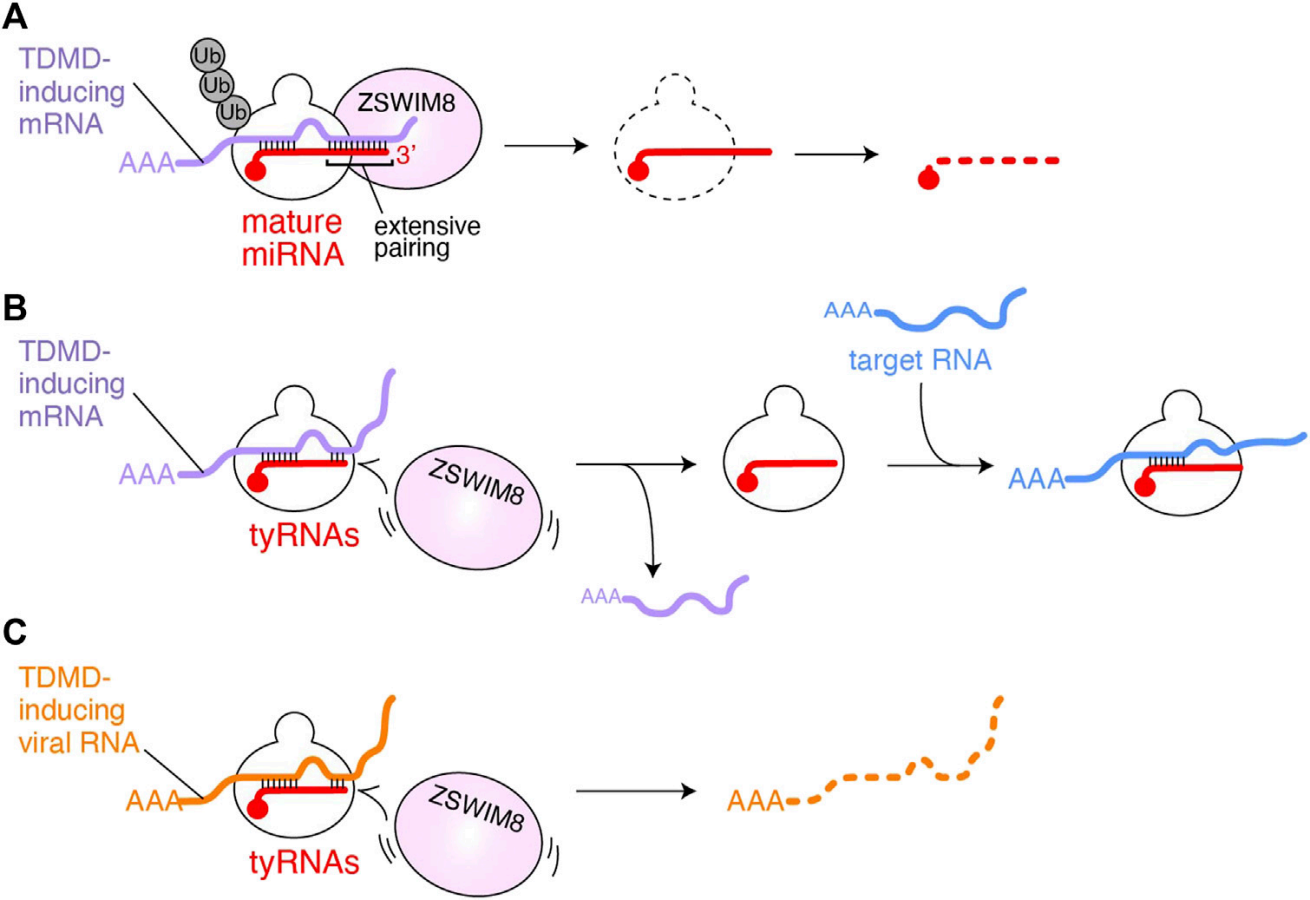
Possible model of tyRNA escaping from TDMD. **(A)** TDMD-inducing mRNA (purple) is base paired with the seed and 3′ region of AGO-associated miRNA (red). The extensive 3′ pairing works as a scaffold for ZSWIM8, followed by polyubiquitination of AGO. As a result, the miRNA is exposed to the cellular RNases and degraded. **(B)** AGO with tyRNAs would fail to recruit ZSWIM8 because it cannot form an extensive pairing with TDMD-inducing mRNAs (purple). Instead, the RISC uses the intact seed to bind to mRNAs (blue) that its full-length mature miRNA targets for gene silencing. **(C)** AGO with tyRNAs escaping from polyubiquitination may degrade TDMD-inducing viral noncoding RNAs (orange).

The target specificity of miRNA-mediated gene silencing primarily relies on the base complementarity between the seed region (g2-g8) of guide RNA and target RNAs (Lewis et al., 2005; Lewis et al., 2003; Zisoulis et al., 2010). Further pairing through the 3′ region of the guide reinforces their interaction (Grimson et al., 2007). Some miRNAs are known to be degraded when their 3′ region is extensively base paired with mRNAs. This target-directed miRNA degradation (TDMD) controls the level of specific miRNAs (de la Mata et al., 2015; Denzler et al., 2016; Haas et al., 2016). In contrast, miRNAs causing gene silencing have mismatches in their 3′ region with target mRNAs, which could explain how miRNAs and their targets avoid excessive TDMD (Ameres et al., 2010). Structural studies revealed that when the RISC propagates the guide-target duplex toward the 3′ end of the guide, topological stress accumulates, thereby releasing the 3′ end from the PAZ domain (Wang et al., 2009; Sheu-Gruttadauria et al., 2019). Therefore, it seems likely that when the guide pairs TDMD-inducing mRNAs, the freed 3′ end becomes accessible to 3′→5′ exonucleases and terminal nucleotidyltransferases which trim and tail the 3′ end, respectively (Fuchs Wightman et al., 2018; Warkocki et al., 2018). Although TDMD has been thought to be accompanied by trimming and tailing of the 3′ end guide RNAs, recent studies reported TDMD independent of these 3′ end modifications (Han et al., 2020; Shi et al., 2020) (Figure 2A). In this model, the RISC has a specific conformation due to the extensive pairing between the guide and TDMD-inducing mRNA and recruits Zinc Finger SWIM-Type Containing 8 (ZSWIM8) to form a cullin-RING ubiquitin ligase complex. As a result, AGO is polyubiquitinated by E2 ligase. After a 26S proteosome degrades the ubiquitinated AGO, the miRNAs are exposed to cellular RNases.

On the contrary, unlike mature miRNAs, tyRNAs, having a short 3′ region, do not appear to extensively pair with TDMD-inducing target RNAs (Figure 2B). Since the seed region of tyRNAs is intact, they would retain the ability to silence gene expression (Kuscu et al., 2018; Li et al., 2009) or like cityRNAs may play distinct roles from that of their mature form (Park et al., 2020). Viruses also exploit TDMD to neutralize the antiviral response of the cells that they infect. Herpesvirus saimiri and Murine cytomegalovirus transcribe their noncoding RNAs, H. saimiri U-rich RNA1 (HSUR1) and m169, respectively, both of which include sequences that extensively pair with the seed and 3′ region of miR-27a and thus trigger TDMD (similar to Figure 2A) (Buck et al., 2010; Cazalla et al., 2010). Since miR-27a is involved in the antiviral response (Marcinowski et al., 2012), the degradation of key cellular miRNAs is beneficial to viral infection and replication. If the infected cells change such miRNAs into tyRNAs, the RISC could escape from virus-induced TDMD, and instead degrade the viral noncoding RNAs (Figure 2C). Therefore, the biosynthesis of tyRNAs may counter the infection as part of the antiviral response.
